# Mapping the evolution of neurofeedback research: a bibliometric analysis of trends and future directions

**DOI:** 10.3389/fnhum.2024.1339444

**Published:** 2024-05-10

**Authors:** Walton Wider, Jasmine Adela Mutang, Bee Seok Chua, Nicholas Tze Ping Pang, Leilei Jiang, Muhammad Ashraf Fauzi, Lester Naces Udang

**Affiliations:** ^1^Faculty of Business and Communications, INTI International University, Nilai, Negeri Sembilan, Malaysia; ^2^Faculty of Psychology and Education, Universiti Malaysia Sabah, Kota Kinabalu, Sabah, Malaysia; ^3^Faculty of Medicine and Health Sciences, Universiti Malaysia Sabah, Kota Kinabalu, Sabah, Malaysia; ^4^Faculty of Education and Liberal Arts, INTI International University, Nilai, Negeri Sembilan, Malaysia; ^5^Faculty of Industrial Management, Universiti Malaysia Pahang Al-Sultan Abdullah, Pekan, Pahang, Malaysia; ^6^Faculty of Liberal Arts, Shinawatra University, Pathumthani, Thailand; ^7^College of Education, University of the Philippines, Diliman, Philippines

**Keywords:** neurofeedback, bibliometrics analysis, web of science, human health, co-word analysis, co-citation analysis

## Abstract

**Introduction:**

This study conducts a bibliometric analysis on neurofeedback research to assess its current state and potential future developments.

**Methods:**

It examined 3,626 journal articles from the Web of Science (WoS) using co-citation and co-word methods.

**Results:**

The co-citation analysis identified three major clusters: “Real-Time fMRI Neurofeedback and Self-Regulation of Brain Activity,” “EEG Neurofeedback and Cognitive Performance Enhancement,” and “Treatment of ADHD Using Neurofeedback.” The co-word analysis highlighted four key clusters: “Neurofeedback in Mental Health Research,” “Brain-Computer Interfaces for Stroke Rehabilitation,” “Neurofeedback for ADHD in Youth,” and “Neural Mechanisms of Emotion and Self-Regulation with Advanced Neuroimaging.

**Discussion:**

This in-depth bibliometric study significantly enhances our understanding of the dynamic field of neurofeedback, indicating its potential in treating ADHD and improving performance. It offers non-invasive, ethical alternatives to conventional psychopharmacology and aligns with the trend toward personalized medicine, suggesting specialized solutions for mental health and rehabilitation as a growing focus in medical practice.

## Introduction

Neurofeedback is also known as EEG biofeedback and brainwave biofeedback (Hellrung et al., 2022). The primary objective of neurofeedback is to modify brain electrical activity, which is the basis for emotional and behavioral processes in the body (Mirifar et al., 2022). It combines electroencephalogram (EEG) capabilities with advances in computer technology and operant conditioning (Swingle and Psych, 2010). Neurofeedback enables the brain to self-identify and adjust or self-regulate its electrical activity through the use of specific treatment procedures that either reward (strengthen) or inhibit (weaken) specific brainwave patterns (Ninaus et al., 2015). Participants can learn to interrupt dysfunctional neurological patterns and create more stable brainwave patterns. A remarkable embodiment of neurofeedback principles can be seen in brain-computer interfaces (BCIs) for motor rehabilitation, particularly after stroke (Sebastián-Romagosa et al., 2020). Remsik et al. (2021) describe the use of brain-computer interfaces (BCIs) for stroke rehabilitation through neurofeedback based on operant conditioning. This approach allows stroke survivors to purposefully control their brain’s sensorimotor rhythms by providing real-time feedback when they generate the desired brain activity. This method facilitates neurological recovery and can significantly improve motor function by reinforcing beneficial neural patterns, helping patients re-learn motor skills damaged by stroke. These advances highlight neurofeedback’s uniqueness and potency as a rehabilitation method, diverging fundamentally from conventional self-regulation and cognitive-behavioral interventions by offering specificity, direct targeting of brain function, and immediate, personalized feedback.

Neurofeedback distinguishes itself from conventional self-regulation and cognitive-behavioral techniques by directly focusing on and altering brain activity. Unlike traditional techniques that primarily aim to adjust thoughts, emotions, or behaviors through subjective means (Zabihiyeganeh et al., 2019; Stran et al., 2020), neurofeedback utilizes a range of imaging modalities including real-time EEG, fMRI, MEG, and NIRS to provide objective, individualized insights into brain function, offering a more precise and data-driven approach to understanding and modifying neural activity (Emmert et al., 2016; Marzbani et al., 2016; Kvamme et al., 2022; Wu et al., 2022; Yagi et al., 2022; Flanagan and Saikia, 2023; Lieberman et al., 2023). This level of specificity enables the focused training of specific brain areas and frequencies that are associated with certain functions or illnesses. This sets it apart from the more general effectiveness of conventional methods (Hammond, 2007). Furthermore, neurofeedback’s immediate feedback loop permits real-time self-modulation of brain activity (Marzbani et al., 2016), contrasting with the delayed feedback or the required conscious efforts associated with conventional therapies. Neurofeedback’s training paradigm is uniquely thorough compared to traditional procedures since it requires several sessions to induce enduring changes in brain function, rather than focusing on short-term effects. Hence, neurofeedback provides a specific, direct technique for improving brain activity and attaining therapeutic aims, substantially distinct from the broader, more generic tactics applied by traditional self-regulation and cognitive-behavioral therapies.

The effects of neurofeedback on cognitive function, with a focus on memory, are based on the principle of operant conditioning and involve informing the subject in real time about the workings of their organism to motivate them to change their behavior (Pérez-Elvira et al., 2021). Neurofeedback is founded on two fundamental principles. First, the EEG accurately reflects observable mental states; the second reason is that these states can be educated (Thompson and Thompson, 2003). The neurofeedback method aims to accomplish two primary goals. The first involves altering a specific brainwave frequency in a region of the participant’s brain that has been linked to their current emotional or behavioral issue (Marzbani et al., 2016). The second objective is to improve the stability and communication of neural networks across the brain and between or within its hemispheres (Sitaram et al., 2017). Neurofeedback restores the brain’s rhythm, timing, frequency, and synchronization, allowing the brain to better coordinate perception, movement, and conscious experience (Farmer, 2002).

EEG neurofeedback systems utilize both operant conditioning and classical (associative) learning principles in the context of motor rehabilitation. Operant conditioning is employed to reinforce desired brain activity patterns associated with motor function. For example, when a patient generates specific brainwave patterns indicative of motor planning or execution, they may receive positive feedback such as auditory or visual cues, encouraging them to continue producing those patterns. Classical (associative) learning is utilized to establish connections between movement-related cues or mental imagery and positive outcomes. For instance, patients might be trained to associate imagining the movement of their limbs with successful motor execution or reduced pain, facilitating motor relearning and rehabilitation. By combining these learning principles, EEG neurofeedback systems can effectively engage both voluntary behavior modification and reflexive response associations, enhancing motor rehabilitation outcomes for patients.

Vernon et al. (2003) asserted that prior research suggests neurofeedback may be effective in treating a variety of early childhood disorders. Including attention-deficit/hyperactivity disorder (ADHD), Asperger’s disorder, learning disability, obsessive-compulsive disorder (OCD), and autism spectrum disorder (ASD) (McVoy et al., 2019; Naeimian et al., 2020; Direito et al., 2021; Riesco-Matías et al., 2021; Zafarmand et al., 2022). Several randomized clinical studies on the use of neurofeedback techniques for ADHD have demonstrated the efficacy of neurofeedback (Gevensleben et al., 2009; Sonuga-Barke et al., 2013; Micoulaud-Franchi et al., 2014; Cortese et al., 2016; Young et al., 2017). Because autistic children frequently exhibit symptoms of attention deficit and hyperactivity, these findings have prompted research into neurofeedback as an alternative treatment for autism (Klöbl et al., 2023). Neurofeedback therapy has also been shown in studies to be effective and beneficial in the treatment of a variety of mental disorders, including anxiety, depression (Wang et al., 2022), sleep disorders (Kolken et al., 2023), headaches (Arina et al., 2022), migraines (Hashemipour and Isfahani Asl, 2022), and other emotional issues (Zotev et al., 2011). It has also been shown to be effective in treating people with organic brain disorders such as cerebral palsy, and seizures (Nigro, 2019). Other studies have shown that neurofeedback has the potential to improve optimal performance in high-level musical performers (Egner and Gruzelier, 2003), dance performance (Raymond et al., 2005), and sports performance (Xiang et al., 2018; de Brito et al., 2022).

## Literature review

Bibliometric analyses have been useful in identifying key research trends and mapping the intellectual structure of neurofeedback-related research. For instance, Rong et al. (2022) and Yao et al. (2022) conducted bibliometric analyses on ASD and quantitative EEG research in neuropsychiatric disorders, revealing the most influential authors, institutions, and countries in the field as well as the most frequently studied brain regions and EEG features. These analyses shed light on the global research status and trends in autism spectrum disorder (ASD) and electroencephalogram (EEG), as well as how neurofeedback can be used as a treatment option, providing valuable insights for researchers and practitioners. In addition, bibliometric evaluations of the publication history and influence of neurofeedback research have been conducted. Onganlar et al. (2021) conducted a comprehensive analysis of neurofeedback articles published between 1975 and 2020, providing a historical overview of publication trends, citation patterns, and research topics. Using bibliometrics and content analysis based on natural language processing, Wang et al. (2022) investigated changes in depression and radiology-related publications, revealing the evolution of research focus in these fields. These analyses provide historical context and emphasize the dynamic nature of neurofeedback research.

Meta-analyses have also been conducted to systematically evaluate the effects of neurofeedback on particular outcomes, in addition to bibliometric analyses. A meta-analysis conducted by Yeh et al. (2022) examined the effects of neurofeedback training on working memory and episodic memory in healthy populations, providing evidence for the cognitive benefits of neurofeedback. In addition, meta-analyses have been conducted to evaluate the efficacy of neurofeedback in treating ADHD, with studies by Arns et al. (2020), Chiu et al. (2022), and Cortese et al. (2016) revealing promising results for improving inattention, impulsivity, and hyperactivity in individuals with ADHD. These meta-analyses provide valuable evidence regarding the potential therapeutic benefits of neurofeedback in specific populations. In addition, empirical research has investigated the efficacy of neurofeedback in treating various neuropsychiatric conditions. For example, Arns et al. (2009) conducted a meta-analysis on the efficacy of neurofeedback for ADHD and found significant improvements in core ADHD symptoms. Russo et al. (2022) conducted a meta-analysis on neurofeedback for anxiety spectrum disorders, revealing promising results for anxiety symptom reduction. These empirical studies shed light on the clinical applications of neurofeedback and support its potential as a treatment for neuropsychiatric disorders.

Additionally, the meta-analyses conducted by Cervera et al. (2018), Nojima et al. (2022), and Vavoulis et al. (2023) jointly emphasize the effectiveness of Brain-Computer Interface (BCI) systems in improving motor recovery after a stroke. BCIs have shown notable enhancements in motor performance by enabling the regulation of sensorimotor rhythms through neurofeedback. These benefits are measured using assessments like the Fugl-Meyer Assessment. The use of BCIs in rehabilitation not only provides a platform for neuroplasticity but also suggests the possibility of functional and structural brain healing. Despite encouraging findings, these studies highlight the demand for more research to enhance BCI technology, optimize training methods, and test the clinical efficacy through bigger, more varied study populations, hoping to secure BCI’s place in the future of neurorehabilitation.

### Present study

The purpose of this investigation is to provide a thorough understanding of the neurofeedback research literature. To the best of the authors’ knowledge, no prior bibliometric study in this area has been conducted. Our study aims to supplement Onganlar et al. (2021) overview of bibliometric analysis because their study only focuses on publication trends, citation patterns, and research topics over time. This study, on the other hand, focuses on examining neurofeedback literature using a co-citation and co-word approach. By utilizing these two bibliometric analyses, this study fills a void by providing insights into past, present, and future research directions. As a result of the specific bibliometric analyses, the following research objectives emerge:

1. To assess significant historical research on neurofeedback using co-citation analysis.
2. To assess emerging trends in neurofeedback using co-word analysis.

## Methods

### Bibliometric approach

Bibliometric techniques are useful for examining the connections between scientific papers and identifying trends and patterns in the evolution of research disciplines (Wider et al., 2023a). Co-citation analysis is the process of identifying two or more documents that were cited in the reference section of a third paper (Bronk et al., 2023). This analysis of co-citation connections across publications enables researchers to identify clusters of frequently cited works related to specific research topics or subdomains (Li et al., 2023). These classifications provide insights into a research field’s intellectual foundation, the evolution of research themes, and the long-term impact of significant works (Donthu et al., 2021; Wider et al., 2023b). Furthermore, co-citation analysis can aid in the identification of prominent authors, institutions, and journals that have contributed to the advancement of a research field (Gao et al., 2022).

Co-word analysis, on the other hand, entails detecting terms or phrases that appear together in the titles, abstracts, or keywords of academic papers (Dhiman et al., 2023). Researchers can uncover clusters of interconnected research subjects, themes, or ideas by studying the co-occurrence patterns of these terms (Lim et al., 2022; Zakaria et al., 2023). These clusters provide useful information about a research domain’s academic interests and intellectual organization. Furthermore, co-word analysis can help identify emerging research topics and trends, as well as track the evolution of research themes over time (Liu et al., 2021; Wider et al., 2023c).

Researchers can investigate the historical, current, and potential future trends in neurofeedback by using bibliometric techniques such as co-citation and co-word analysis. Neurofeedback research has shown potential benefits in improving cognitive performance, treating neurological disorders, and addressing mental health issues (Loriette et al., 2021). Bibliometric analysis can help identify the most important or highly cited works related to these applications. Researchers can identify the most influential works in neurofeedback research and track their evolution over time by examining co-citation patterns and co-occurring terms. Furthermore, co-word analysis can help identify emerging subjects or trends in neurofeedback research, such as its potential as a powerful therapeutic tool. In conclusion, bibliometric techniques assist researchers in gaining a thorough understanding of the potential benefits of neurofeedback, its progression over time, and its possible future trajectory.

### Search string

The search string used in this bibliometric investigation is detailed in Table 1. The topic search (TS) feature of the Web of Science (WOS) database was used to limit terms to titles, abstracts, and keywords. The search term “neurofeedback” covered articles from 1989 to 2023. The search took place on April 6, 2023. The WOS database is well-known for its high quality and comprehensiveness, making it an excellent choice for bibliometric research. It is the world’s oldest, most widely used, and most trustworthy research publication and citation database, providing selective, balanced, and comprehensive coverage of the world’s leading research from over 34,000 journals (Birkle et al., 2020). Eugene Garfield founded Web of Science in 1964 as the Science Citation Index, and it has since expanded its scope to cover a wide range of disciplines.

**Table 1:** Search string, inclusion, and exclusion criteria.

Wos database	ALL
Time Period	Up to April 6th, 2023
Search field	Topic
Search keywords	“neurofeedback”
Citation Topics Meso	ALL
Document Type	ALL
Languages	English

**Figure 1:**
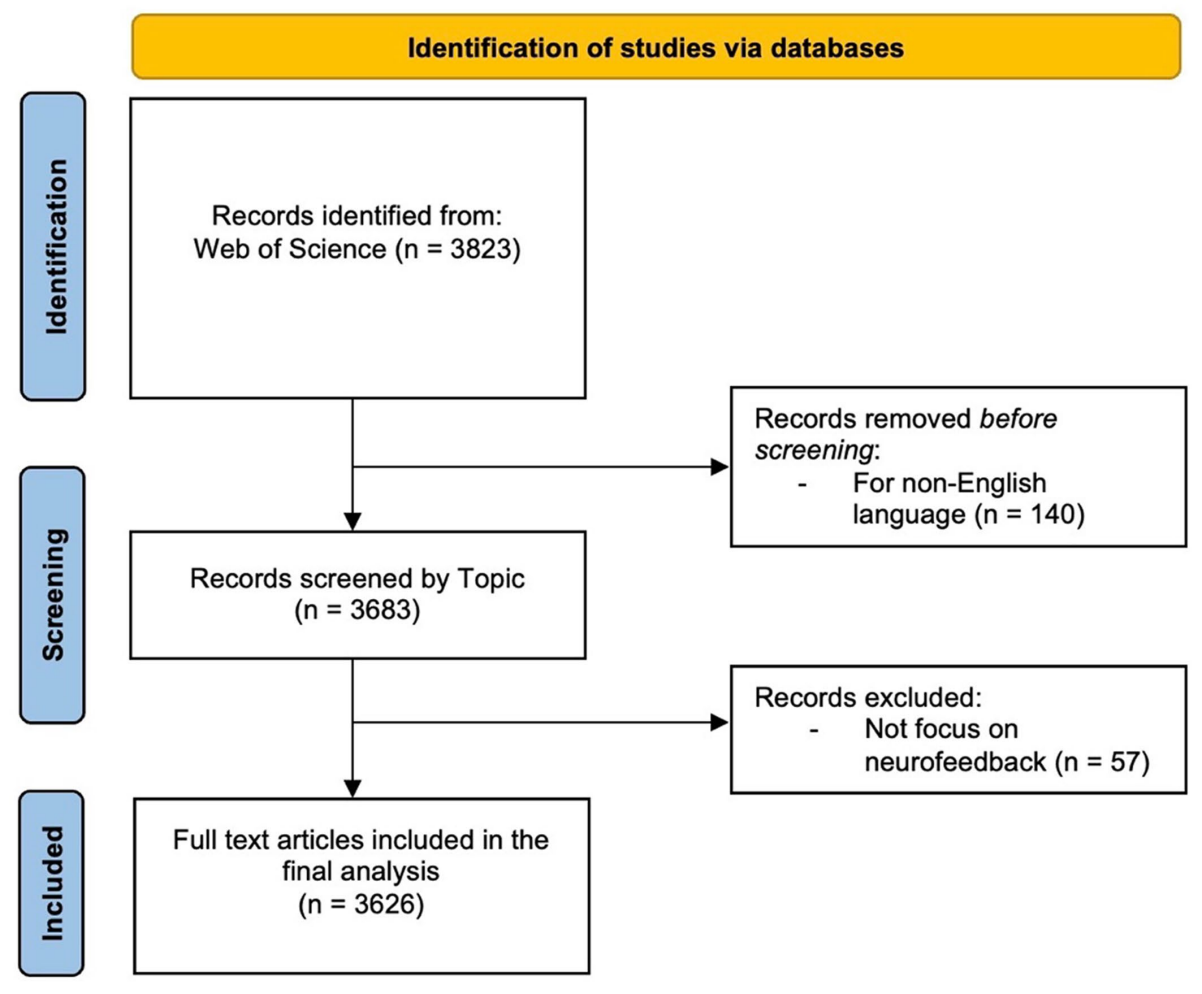
Flowchart illustrating the search results following the modified PRISMA standard.

The search was performed in the WOS Database, a large academic database that indexes conference proceedings, scientific journals, and books. The “Search Field” section outlines the parameters that confined the search to the subject area, encompassing the title, abstract, and keywords of a publication. The search period was extended until April 6th, 2023 in order to include all available publications in the results. To ensure data integrity, all publications were checked for inconsistencies and duplicates prior to conducting the bibliometric analysis (Linnenluecke et al., 2020). Because the citation topics were set to “ALL,” the search results included all publications’ topics, regardless of their specific research focus. Articles, reviews, editorials, and conference proceedings were all included in the “ALL” document type. The search was restricted to publications written in English, which is a widely used language in scientific communication. This restriction ensured that the findings were accessible to a wide range of readers and researchers. Table 1 shows the inclusion and exclusion criteria for this review. Based on these criteria, the screening process retained 3,626 articles (Figure 1). The article selection process was conducted in accordance with the Preferred Reporting Items for Systematic Reviews and Meta-Analyses (PRISMA) methodology (Page et al., 2021).

## Results and discussion

### Publication trends and descriptive analysis

**Figure 2:**
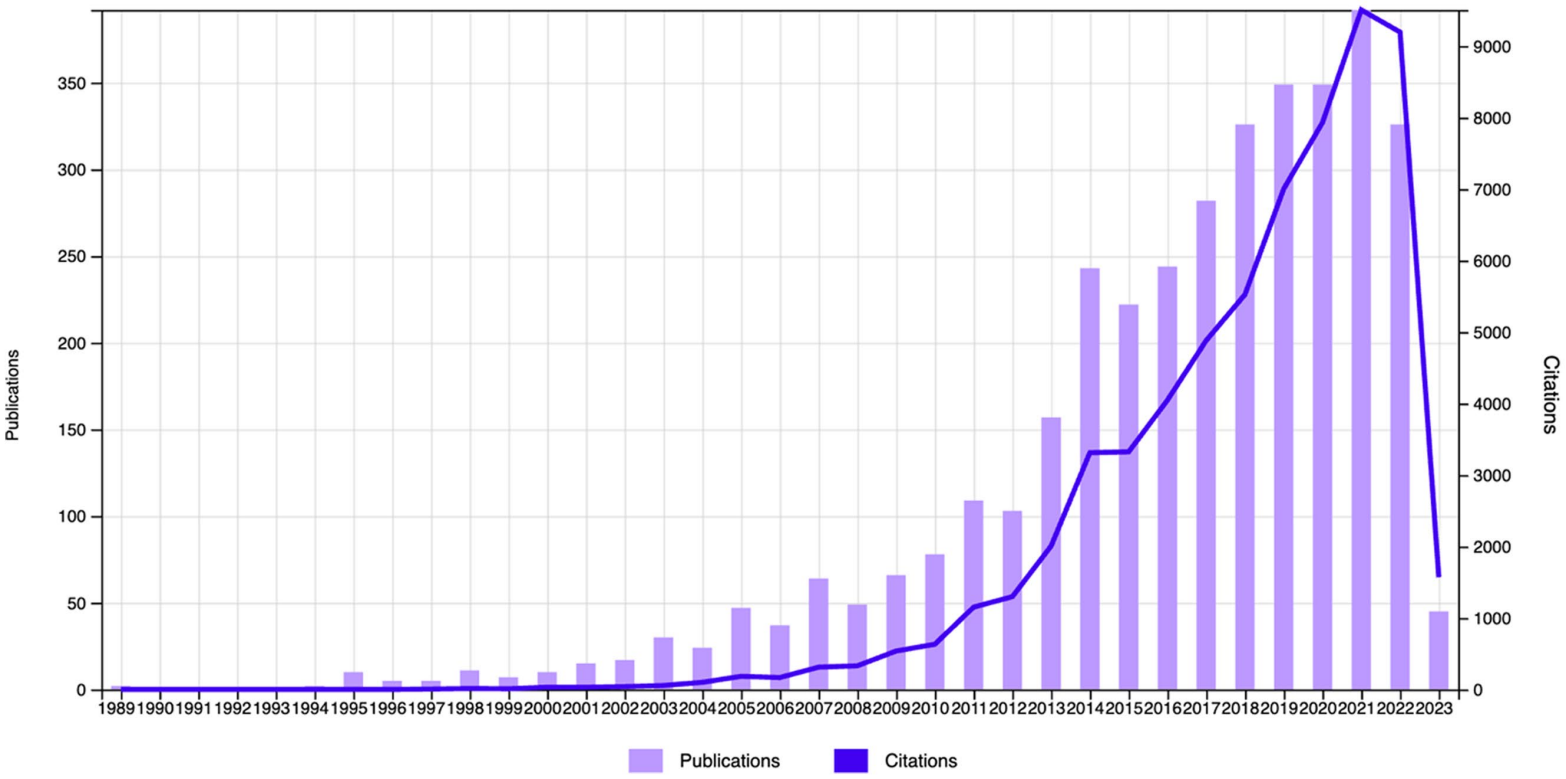
Number of articles and citations from 1989 to April 6, 2023.

The Web of Science (WOS) database revealed 63,195 citations linked to the selected studies (*N* = 3,626), with a reduction to 33,1,234 when self-citations were excluded. These articles had an H-index of 111 and an average citation count of 17.43 per paper. The body of 3,626 articles reflects a growing interest in neurofeedback research. Although the inaugural paper on neurofeedback appeared in 1989, it wasn’t until 1994 that significant scholarly contributions were noted. Post-1994, publication frequency has surged exponentially. Growth was modest before the 21st century, but from 2000 to 2021, there was a marked escalation in the number of publications, soaring from 10 in 2000 to 392 in 2021, representing a substantial increase over two decades. In 2022, however, there was a slight dip in publications, decreasing to 326. It is anticipated that scholarly focus on neurofeedback will continue to ascend in the forthcoming years. Figure 2 illustrates the trajectory of published articles, and their citation counts from 1989 to 2023.

### Co-citation analysis

**Figure 3:**
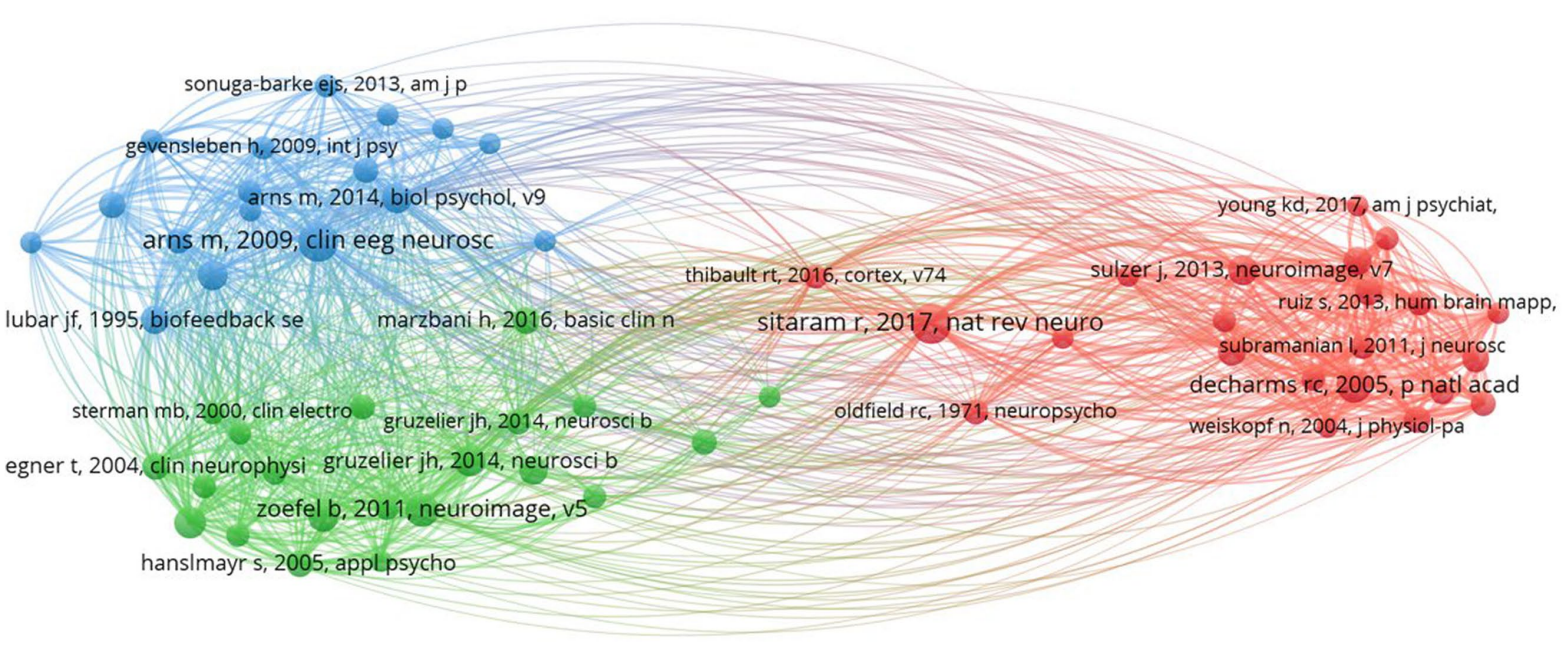
Co-citation analysis.

In our co-citation analysis, we set a citation threshold of 86, meaning that only references cited together 86 times or more were included. This methodology led to the identification of 60 references that met or exceeded this co-citation frequency threshold, ensuring that our analysis concentrated on the most significant and relevant themes within the scientific literature. This threshold was determined through a series of tests aimed at ensuring the clusters identified were stable and accurately represented relevant themes. The optimal threshold was established after experimenting with various levels, specifically 54, 55, 57, 58, 59, 61, and 63. Table 2 displays the top 10 co-cited references with the highest total link strength. The study by Arns et al. (2009) received 284 citations, followed by DeCharms et al. (2005) with 228 citations, and Zoefel et al. (2011) with 200 citations. Figure 3 presents a network analysis of neurofeedback research, based on the cited references.

**Table 2:** Top 10 documents with the highest co-citation and total link strength.

No.	Documents	Citation	Total link strength
1	Arns, M., De Ridder, S., Strehl, U., Breteler, M., & Coenen, A. (2009). Efficacy of neurofeedback treatment in ADHD: the effects on inattention, impulsivity and hyperactivity: a meta-analysis. *Clinical EEG and neuroscience, 40*(3), 180–189.	284	1,505
2	DeCharms, R. C., Maeda, F., Glover, G. H., Ludlow, D., Pauly, J. M., Soneji, D., & Mackey, S. C. (2005). Control over brain activation and pain learned by using real-time functional MRI. *Proceedings of the National Academy of Sciences*, *102*(51), 18,626–18,631.	228	1,334
3	Zoefel, B., Huster, R. J., & Herrmann, C. S. (2011). Neurofeedback training of the upper alpha frequency band in EEG improves cognitive performance. *Neuroimage*, *54*(2), 1,427–1,431.	200	1,145
4	Linden, D. E., Habes, I., Johnston, S. J., Linden, S., Tatineni, R., Subramanian, L., & Goebel, R. (2012). Real-time self-regulation of emotion networks in patients with depression. *PloS one*, *7*(6), e38115.	157	1,081
5	Sitaram, R., Ros, T., Stoeckel, L., Haller, S., Scharnowski, F., Lewis-Peacock, J., & Sulzer, J. (2017). Closed-loop brain training: the science of neurofeedback. *Nature Reviews Neuroscience*, *18*(2), 86–100.	284	1,081
6	Gruzelier, J. H. (2014a). EEG-neurofeedback for optimizing performance. I: A review of cognitive and affective outcome in healthy participants. *Neuroscience & Biobehavioral Reviews*, *44*, 124–141.	185	1,013
7	Vernon, D., Egner, T., Cooper, N., Compton, T., Neilands, C., Sheri, A., & Gruzelier, J. (2003). The effect of training distinct neurofeedback protocols on aspects of cognitive performance. *International journal of psychophysiology*, *47*(1), 75–85.	193	1,008
8	Sulzer, J., Haller, S., Scharnowski, F., Weiskopf, N., Birbaumer, N., Blefari, M. L., & Sitaram, R. (2013). Real-time fMRI neurofeedback: progress and challenges. *Neuroimage*, *76*, 386–399.	174	991
9	Caria, A., Veit, R., Sitaram, R., Lotze, M., Weiskopf, N., Grodd, W., & Birbaumer, N. (2007). Regulation of anterior insular cortex activity using real-time fMRI. *Neuroimage*, *35*(3), 1,238–1,246.	130	946
10	Zotev, V., Krueger, F., Phillips, R., Alvarez, R. P., Simmons, W. K., Bellgowan, P., & Bodurka, J. (2011). Self-regulation of amygdala activation using real-time fMRI neurofeedback. *PloS one*, *6*(9), e24522.	135	926

Source: Interpretation by authors.

Through the examination of co-citations, it becomes evident that there are three distinct clusters, each centered around a specific theme. These clusters represent groups of related items that share a common theme. Related articles are organized into clusters, indicated by nodes of matching colors (Dong et al., 2023). Below is the description of each cluster and its corresponding label.

- Cluster 1 (red) is comprised of 22 publications titled “**Real-Time fMRI neurofeedback and self-regulation of brain activity**.” Neurofeedback based on real-time functional magnetic resonance imaging (fMRI) has emerged as a powerful tool for understanding and modulating brain activity, with significant implications for mental health and cognitive enhancement (Martz et al., 2020; Direito et al., 2021). This collection of research articles looks into the methodologies, applications, and challenges of real-time fMRI neurofeedback, with a particular emphasis on brain activity self-regulation. Birbaumer et al. (2013) and Sitaram et al. (2017) provide comprehensive reviews of neurofeedback science, highlighting the potential of closed-loop brain training in treating a variety of neurological and psychiatric disorders. These studies highlight the significance of learning how to regulate brain metabolism as well as the potential of neurofeedback as a non-invasive intervention. Caria et al. (2007, 2010) investigate the regulation of anterior insular cortex activity and show that volitional control over this area modifies responses to aversive stimuli. These findings could help us understand and treat anxiety and other emotional disorders. Similarly, DeCharms et al. (2004, 2005) demonstrate that learned regulation of spatially localized brain activation can lead to improved pain perception control. Shibata et al. (2011) present a novel approach to perceptual learning based on decoded fMRI neurofeedback, demonstrating that learning can be induced without the need for stimulus presentation. This study demonstrates the potential of real-time fMRI neurofeedback for improving cognitive performance across multiple domains. Young et al. (2014, 2017) investigate the use of real-time fMRI neurofeedback in the treatment of major depressive disorder, demonstrating that training amygdala activity can result in significant improvements in symptoms and autobiographical memory recall. These findings highlight neurofeedback’s therapeutic potential for mental health conditions. Sulzer et al. (2013), Weiskopf (2012) and Weiskopf et al. (2003, 2004) investigate the methodologies and exemplary data associated with real-time fMRI neurofeedback, emphasizing the potential for physiological self-regulation of regional brain activity. This research focuses on the technical aspects and challenges of this rapidly evolving field. Finally, Zotev et al. (2011) investigate amygdala activation self-regulation, bolstering the potential of real-time fMRI neurofeedback in treating emotional disorders and improving emotional control. In summary, this cluster demonstrates the efficacy and potential of real-time fMRI neurofeedback in understanding and modulating brain activity, with important implications for mental health, cognitive enhancement, and the future of neuroscience.
- Cluster 2 (green) contains 21 publications titled “**EEG-Neurofeedback and Cognitive Performance Enhancement**.” The studies in this cluster are concerned with the effects of EEG-neurofeedback on cognitive performance as well as the methodologies involved. EEG-neurofeedback, a type of biofeedback, entails measuring and providing real-time feedback on EEG activity to help people learn self-regulation of brain activity and improve cognitive performance (Marzbani et al., 2016; Ramalingam et al., 2023). This has been explored in healthy participants (Gruzelier, 2014a) and those with neurological disorders such as epilepsy (Sterman and Egner, 2006). Gruzelier (2014a,b) provides comprehensive reviews on performance optimization using EEG-neurofeedback, with an emphasis on methodological and theoretical considerations. Gruzelier (2014a) emphasizes the beneficial effects on cognition and affect in healthy participants, whereas Gruzelier (2014b) discusses the importance of effective protocols as well as the role of individual differences. Several studies have been conducted to examine the effect of neurofeedback training on specific EEG frequency bands. Both Zoefel et al. (2011) and Hanslmayr et al. (2005) show that increasing upper alpha power via neurofeedback improves cognitive performance. Klimesch (1999) also discovered that EEG alpha and theta oscillations reflect cognitive and memory performance. Egner and Gruzelier (2001, 2004), on the other hand, concentrate on the low beta band components, reporting frequency-specific effects on attention and event-related brain potentials. Vernon et al. (2003) investigate the effect of different neurofeedback protocols on cognitive performance, whereas Delorme and Makeig (2004) present EEGLAB, an open-source toolbox for analyzing single-trial EEG dynamics, including independent component analysis. These tools are critical for researchers to analyze and comprehend the underlying neural processes associated with neurofeedback. Egner and Gruzelier (2003) demonstrate that slow-wave EEG modulation improves musical performance, addressing the ecological validity of neurofeedback. This study emphasizes the practicality of neurofeedback training. Finally, Vernon (2005) assesses the evidence for neurofeedback training’s ability to improve performance and emphasizes the need for additional research in order to draw firm conclusions. In summary, the studies in this cluster investigate the ability of EEG-neurofeedback to improve cognitive performance across multiple domains. They emphasize the relevance of specific frequency bands and the ecological validity of neurofeedback training, as well as methodological considerations, effective protocols, and individual differences.
- Cluster 3 (blue) contains 17 publications with the title “**Treatment of attention-deficit/hyperactivity disorder (ADHD) using neurofeedback**.” This article collection looks into the efficacy, outcomes, and potential of neurofeedback as an alternative or complementary approach to managing ADHD symptoms in children and adolescents. The comparison of neurofeedback to traditional pharmacological treatments, such as methylphenidate, is a recurring theme in these articles (Fuchs et al., 2003; Sonuga-Barke et al., 2013). This cluster includes multiple meta-analysis that synthesize findings from various studies to provide a comprehensive understanding of neurofeedback’s effectiveness (Arns et al., 2009; Cortese et al., 2016; Van Doren et al., 2019). These meta-analysis show that neurofeedback training has a positive effect on ADHD symptoms and that the effects last. Several articles investigate specific neurofeedback techniques, such as slow cortical potential training (Heinrich et al., 2004; Strehl et al., 2006), and investigate the underlying neurophysiological effects of these treatments (Gevensleben et al., 2009). These studies contribute to a better understanding of how neurofeedback alters brain function in ADHD patients to improve attention, impulsivity, and hyperactivity. Furthermore, some articles provide critical assessments of neurofeedback research and discuss the difficulties in determining its efficacy (Arns et al., 2014). They emphasize the importance of methodologically rigorous studies and long-term follow-ups in order to establish neurofeedback’s clinical utility as a viable ADHD treatment option. This cluster exemplifies the growing interest in neurofeedback as a non-pharmacological treatment for ADHD, highlighting both its potential benefits and limitations. This cluster contributes to a more comprehensive understanding of neurofeedback’s role in managing ADHD symptoms, as well as the importance of ongoing research in this area.

Table 3 presents a summary of co-citation analysis on neurofeedback with the clusters’ number, color, labels, number of publications and representative publications.

**Table 3:** Co-citation clusters on neurofeedback.

Cluster	Cluster label	Number of publications	Representative publications
1 (red)	Real-Time fMRI neurofeedback and self-regulation of brain activity	22	Birbaumer et al. (2013); Caria et al. (2007); DeCharms et al. (2005); Shibata et al. (2011); Sitaram et al. (2017); Young et al., 2014; Weiskopf (2012); Sulzer et al. (2013); Zotev et al. (2011)
2 (Green)	EEG-Neurofeedback and Cognitive Performance Enhancement	21	Gruzelier (2014a,b); Zoefel et al. (2011); Klimesch (1999); Hanslmayr et al. (2005); Vernon et al. (2003); Egner and Gruzelier (2001, 2003, 2004); Vernon (2005); Marzbani et al. (2016); Delorme and Makeig (2004); Sterman and Egner (2006).
3 (Blue)	Treatment of attention-deficit/hyperactivity disorder (ADHD) using neurofeedback	17	Association, A. P (2013); Arns et al. (2009); Arns et al. (2014); Fuchs et al. (2003); Lubar et al. (1995); Heinrich et al. (2004), Heinrich et al. (2007); Cortese et al. (2016); Van Doren et al. (2019); Strehl et al. (2006); Sonuga-Barke et al. (2013), Gevensleben et al. (2009).

Source: Interpretation by authors from VOSviewer analysis.

### Co-occurrence of keyword

In our co-word analysis, we identified a total of 63 keywords, with a minimum occurrence threshold set at 61. This threshold was crucial in ensuring that our analysis focused on the most frequently occurring and relevant keywords within our dataset, thereby highlighting key trends and areas of focus in the scientific literature. To determine the most effective threshold for our analysis, we conducted a series of tests using various levels, specifically 62, 64, 65, 66, and 67. This rigorous testing process helped us to identify a threshold that accurately captures the core themes and facilitates a stable and meaningful analysis of the relationships between keywords in our study. The co-word analysis revealed that the most frequently used keyword was “neurofeedback” (1,684 occurrences), followed by “EEG” (617 occurrences) and “ADHD” (378 occurrences). Table 4 displays the top 15 co-word analysis keywords.

**Table 4:** The 15 most frequent keywords in the keyword co-occurrence analysis.

Rank	Keyword	Occurrences	Total link strength
1.	Neurofeedback	1,684	5,285
2.	EEG	617	2,142
3.	ADHD	378	1,685
4.	Children	341	1,496
5.	Attention	359	1,410
6.	Biofeedback	342	1,353
7.	Self-regulation	263	1,246
8.	Performance	276	1,167
9.	Brain	302	1,126
10.	Real-time fMRI	239	961
11.	Attention-deficit/hyperactivity disorder	198	894
12.	fMRI	210	836
13.	Brain-computer interface	200	788
14.	Motor imagery	176	737
15.	Slow cortical potentials	133	703

**Figure 4:**
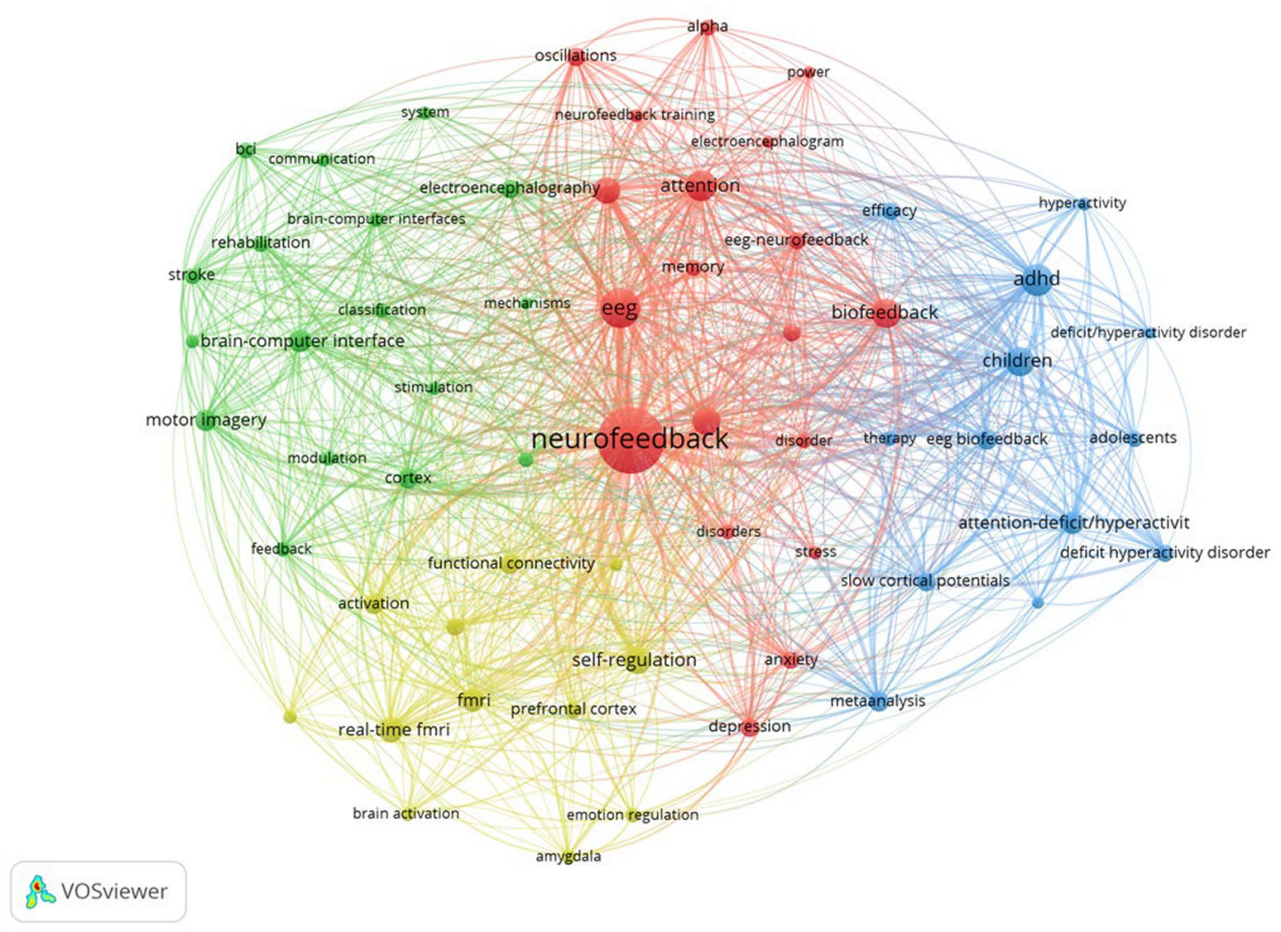
Co-word analysis of neurofeedback research.

Following that, Figure 4 depicts the network structure of keyword co-occurrence. The diagram depicts four distinct clusters that appear to be related. Each cluster was examined and expanded upon as follows:

- Cluster 1 (Red): This cluster comprises a total of 19 keywords and is titled “neurofeedback and mental health research.” This cluster demonstrates the growing importance of neurofeedback techniques, such as EEG-neurofeedback and biofeedback, in the assessment and treatment of mental health disorders such as anxiety, depression, and attention-related issues (Young et al., 2017; Hey, 2020; Taschereau-Dumouchel et al., 2022). Keywords such as “alpha,” “oscillations,” and “power” highlight the emphasis on specific brainwave patterns and their potential role in the manifestation and treatment of these disorders (Klimesch, 1999; Egner and Gruzelier, 2003; Hanslmayr et al., 2005; Zoefel et al., 2011; Perera et al., 2022). Furthermore, the cluster emphasizes the importance of working memory and cognitive performance, indicating the growing interest in using neurofeedback training to improve overall brain function. Researchers are increasingly interested in using neurofeedback to improve cognitive performance, memory, and attention in healthy individuals, in addition to addressing mental health issues (Egner and Gruzelier, 2001; Gruzelier, 2014a; Marlats et al., 2020; Da Silva and De Souza, 2021; Moradi et al., 2022). Based on this cluster, future trends in neurofeedback research are expected to explore the connections between brain oscillations, mental health, and cognitive performance. Researchers may develop more targeted neurofeedback protocols to address specific disorders or enhance specific cognitive abilities as our understanding of the brain’s intricate processes grows. Furthermore, advances in EEG and biofeedback technology may result in more accessible and personalized neurofeedback training methods, allowing a broader range of people to benefit from these interventions. Finally, the cluster formed around these keywords reflects neurofeedback’s growing importance in the research and treatment of mental health disorders and cognitive enhancement. Future trends in this field are likely to focus on improving neurofeedback training methods and making these interventions more accessible to a larger population.
- Cluster 2 (green): There are 17 keywords in this cluster. Based on the keywords, a cluster reveals a significant research focus on the “**development and application of brain-computer interfaces (BCIs) for stroke patient rehabilitation**.” This cluster is concerned with the use of BCIs and their underlying mechanisms, such as electroencephalography (EEG) and transcranial magnetic stimulation (TMS), for rehabilitation purposes. BCIs allow direct communication between the brain and external devices, allowing neural activity to be translated into actionable commands (Shih et al., 2012; Kosal and Putney, 2022). One critical application of this technology is in the field of motor recovery and rehabilitation, particularly for people who have had a stroke (Cervera et al., 2018; Fu et al., 2023). In this context, the study looks into the use of motor imagery techniques, which involve mental rehearsal of motor actions without physical execution, in conjunction with BCIs (Vavoulis et al., 2023). EEG detects neural activity associated with motor imagery, and by modulating this activity, stroke patients can regain control of their motor functions (Liao et al., 2023). TMS is also used as a non-invasive brain stimulation method to facilitate cortical reorganization and improve the efficacy of rehabilitation (Naro and Calabrò, 2022). Furthermore, the cluster emphasizes the significance of feedback and classification systems in the development of effective BCI-based rehabilitation programs (Gao et al., 2022). These systems enable the accurate interpretation and real-time adjustment of the user’s neural activity, allowing for a more personalized and adaptive approach to rehabilitation. Future trends in this cluster are likely to focus on refining and expanding BCI technologies for stroke rehabilitation, with an emphasis on increasing the accuracy and reliability of classification and communication systems (Al-Qazzaz et al., 2023). Furthermore, the incorporation of machine learning and artificial intelligence techniques may aid in the development of more sophisticated and adaptive BCIs (Barnova et al., 2023). Ultimately, these advances could lead to more effective and personalized rehabilitation interventions, significantly improving the quality of life and recovery outcomes for stroke patients.
- Cluster 3 (Blue): There are 13 keywords in this cluster. Based on the keywords, one possible cluster is “**neurofeedback for ADHD in children and adolescents**.” This cluster demonstrates a strong emphasis on understanding and treating ADHD symptoms through the use of EEG biofeedback and slow cortical potentials as therapeutic modalities. The keywords “ADHD,” “adolescents,” “attention-deficit/hyperactivity disorder,” “children,” “deficit hyperactivity disorder,” and “deficit/hyperactivity disorder” highlight the population and condition under investigation. Keywords such as “EEG biofeedback,” “slow cortical potentials,” “therapy,” and “symptoms,” on the other hand, indicate the research’s methodological and therapeutic aspects. The terms “efficacy,” “meta-analysis,” and “hyperactivity” indicate a growing interest in assessing the efficacy of these therapeutic approaches in managing ADHD symptoms, particularly hyperactivity. The presence of “meta-analysis” within this cluster indicates that researchers are synthesizing the findings from multiple studies to obtain a more comprehensive understanding of the efficacy of these interventions (Micoulaud-Franchi et al., 2014; Cortese et al., 2016; Van Doren et al., 2019; Riesco-Matías et al., 2021; Louthrenoo et al., 2022; Kuznetsova et al., 2023). Based on this cluster, future trends in ADHD research and treatment may include a greater focus on neurofeedback techniques such as EEG biofeedback and slow cortical potentials to improve the efficacy of ADHD interventions for children and adolescents (Choudhury et al., 2023). Researchers could concentrate on developing personalized neurofeedback protocols that are tailored to individual needs in order to improve treatment outcomes (Ma et al., 2023; Zhang et al., 2023). Furthermore, there may be an increased interest in researching the long-term effects of these therapies, as well as their potential to reduce or even eliminate the need for pharmacological interventions in some cases (Sibley et al., 2023). Moreover, the integration of new technologies and methods, such as machine learning and real-time brain imaging, could help improve the accuracy and effectiveness of neurofeedback interventions (Haugg et al., 2020; Singh et al., 2022; Taschereau-Dumouchel et al., 2022). This would allow for more targeted targeting of brain regions and neural networks linked to ADHD symptoms. Overall, this cluster points to a future trend in ADHD research that focuses on the development and optimization of novel, non-invasive, and personalized neurofeedback therapies for children and adolescents.
- Cluster 4 (Yellow): There are 12 keywords in this cluster. One possible cluster based on the keywords is “**neural mechanisms of emotion and self-regulation using advanced neuroimaging**.” The cluster of keywords reflects a strong focus on brain function and connectivity research, particularly concerning emotional regulation and self-regulation processes. This cluster indicates a growing interest in studying the neural underpinnings of emotion regulation and self-regulation using advanced neuroimaging techniques such as functional Magnetic Resonance Imaging (fMRI) and real-time fMRI (Zhu et al., 2019; Mathiak and Keller, 2021; Taschereau-Dumouchel et al., 2022; Zotev et al., 2023). This cluster’s connections show an interaction between brain regions, particularly the amygdala and the prefrontal cortex, in modulating emotional responses and self-regulation processes (Lowe et al., 2020; Drigas and Mitsea, 2021; Janet et al., 2023). The amygdala is well-known for its role in emotion processing, particularly fear and anxiety (Šimić et al., 2021), whereas the prefrontal cortex is associated with higher-order cognitive functions and executive control (Friedman and Robbins, 2022). This cluster’s functional connectivity research emphasizes the importance of interactions between these regions in emotion management and self-regulation. More in-depth studies of the dynamic interactions between various brain regions associated with emotion regulation and self-regulation are likely in the future (Yang et al., 2020). This could include creating more advanced real-time fMRI techniques and analysis methods to better understand the temporal and spatial patterns of brain activation and connectivity during these processes (Warbrick, 2022). Furthermore, researchers may investigate the potential of neurofeedback and other neuromodulation techniques to improve emotion regulation and self-regulation by targeting specific brain regions and networks (Barreiros et al., 2019; Melnikov, 2021; James and Duarte, 2023). This could result in the development of novel therapeutic interventions for people suffering from emotional dysregulation, anxiety, depression, or other mental health issues. Furthermore, interdisciplinary research that integrates insights from psychology, psychiatry, and neuroscience may benefit the field by generating a more comprehensive understanding of the neural mechanisms underlying emotion regulation and self-regulation.

The co-word analysis of neurofeedback research is summarized in Table 5, providing information on cluster number, color, labels, number of keywords, and representative keywords.

**Table 5:** Co-word analysis on neurofeedback research.

Cluster No and color	Cluster label	Number of keywords	Representative Keywords
1 (red)	Neurofeedback and mental health research	19	“alpha,” “anxiety,” “attention,” “biofeedback,” “brain,” “depression,” “disorder,” “disorders,” “eeg,” “eeg-neurofeedback,” “electroencephalogram,” “memory,” “neurofeedback,” “neurofeedback training,” “oscillations,” “performance,” “power,” “stress,” “working-memory.”
2 (green)	Development and application of brain-computer interfaces (BCIs) for stroke patient rehabilitation	17	“BCI,” “brain-computer interface,” “brain-computer interfaces,” “classification,” “communication,” “cortex,” “electroencephalography,” “feedback,” “mechanisms,” “modulation,” “motor imagery,” “recovery,” “rehabilitation,” “stimulation,” “stroke,” “system,” “transcranial magnetic stimulation.”
3 (blue)	Neurofeedback for ADHD in children and adolescents	13	“ADHD,” “adolescents,” “attention-deficit/hyperactivity-disorder,” “children,” “deficit hyperactivity disorder,” “deficit/hyperactivity disorder,” “EEG biofeedback,” “efficacy,” “hyperactivity,” “metaanalysis,” “slow cortical potentials,” “symptoms,” “therapy.”
4 (yellow)	Neural mechanisms of emotion and self-regulation using advanced neuroimaging.	12	“activation,” “amygdala,” “brain activation,” “brain activity,” “connectivity,” “emotion regulation,” “fMRI,” “functional connectivity,” “functional MRI,” “prefrontal cortex,” “real-time fMRI,” “self-regulation.”

Source: Interpretation by authors from VOSviewer analysis.

### Implications

This bibliometric study has multiple key clinical implications. Attention deficit hyperactivity disorder is a growing secondary pandemic in the developed world and has been partially exacerbated by the increasing amount of gadget use and consequent Internet and smartphone addiction issues that have emerged. There is hence higher recourse to “urge surfing” using mobile devices, which presents a double whammy for ADHD sufferers. Neurofeedback training has previously been regarded to be in its infancy, but this bibliometric study suggests through the network of keywords and authors that there is much literature of reasonable quality that can be referred to inform the creation of research-grounded, structured protocols as a promising new frontier of treatment for ADHD.

It is essential to acknowledge the significant contributions of neurofeedback to research, highlighting its role as a valuable tool for monitoring brain activity in real-time. Compared to other brain imaging modalities such as fMRI and PET, neurofeedback—often based on EEG—is particularly advantageous due to its non-invasiveness, affordability, and high temporal resolution. These characteristics make it well-suited for providing real-time feedback during neurofeedback interventions, allowing for immediate adjustment and optimization of treatment protocols. This gives it an edge and acts as an essential tool for monitoring real-time brain activity during neurofeedback interventions. Consequently, it enhances our understanding of the mechanisms involved in ADHD treatments which allows researchers and clinicians to customize interventions and evaluate the effectiveness of treatments with accuracy by analyzing variations in brainwave patterns.

Furthermore, the incorporation of transcranial magnetic stimulation (TMS) as a metric of results enhances our comprehension of neurofeedback interventions. TMS acts as a biomarker for enhanced motor function and offers valuable neurophysiological information about corticomuscular excitability. This information deepens our comprehension of the neural processes involved in neurofeedback interventions and can be used to complement behavioral outcomes. The utilization of both neuroimaging and neuromodulatory techniques in neurofeedback research demonstrates its multidisciplinary nature and its ability to improve treatment outcomes for ADHD and potentially other neurological disorders.

In addition to treating ADHD, neurofeedback and biofeedback show great promise in the emerging clinical fields of performance enhancement, especially in sports and occupational psychiatry. The use of neurofeedback will increase clinicians’ repertoire as they can then provide care options that are not invasive, that do not involve the ethical dilemmas of using psychopharmacology and consequent maleficence via unacceptable side effect profiles, while potentially inducing lasting changes in brainwave structure rather than merely symptomatic relief.

### Limitations and conclusion

In conclusion, this bibliometric study demonstrates that there is high potential to grow for neurofeedback and biofeedback as a branch of medical practice. There is already much evidence extant for the role of neurofeedback in stroke and rehabilitation medicine. It now appears to show promise too in the emerging fields of ADHD and performance enhancement, as well as being suitable as a non-invasive treatment modality for general mental health wellness. This bodes well as we move into an age of personalized and precision medicine, where we do not offer one-size-fits-all solutions that offer a broad-based but non-specific treatment for primary and tertiary prevention of mental health disorders.

## Data availability statement

The raw data supporting the conclusions of this article will be made available by the authors, without undue reservation.

## Author contributions

WW: Conceptualization, Data curation, Formal analysis, Funding acquisition, Investigation, Methodology, Project administration, Resources, Software, Supervision, Validation, Visualization, Writing – original draft. JM: Conceptualization, Resources, Validation, Visualization, Writing – review & editing. BC: Conceptualization, Resources, Visualization, Writing – review & editing. NP: Writing – review & editing. MF: Methodology, Validation, Visualization, Writing – review & editing. LU: Funding acquisition, Writing – review & editing. LJ: Writing – review & editing.
